# The role of anti-IgE (omalizumab/Xolair) in the management of severe recalcitrant paediatric atopic eczema (ADAPT): statistical analysis plan

**DOI:** 10.1186/s13063-017-1976-6

**Published:** 2017-05-23

**Authors:** Tao Chen, Susan Chan, Gideon Lack, Suzie Cro, Victoria R. Cornelius

**Affiliations:** 1 Department of Clinical Sciences, Liverpool School of Tropical Medicine, Liverpool, L3 5QA UK; 2 Guy’s and St. Thomas’ NHS Foundation Trust, Westminster Bridge Road, London, SE1 7EH UK; 3 King’s College London, King’s Health Partners, Asthma-UK Centre in Allergic Mechanisms of Asthma, Allergy and Respiratory Science, Guy’s Hospital, London, SE1 9RT UK; 4 Imperial Clinical Trials Unit, School of Public Health, Imperial College London, Stadium House, 68 Wood Lane, London, W12 7RH UK

**Keywords:** Statistical analysis plan, Eczema, Paediatric, Atopic dermatitis, Anti-IgE, Omalizumab, Randomised controlled trial, Xolair

## Abstract

**Background:**

The Atopic Dermatitis Anti-IgE Paediatric Trial (ADAPT) is a trial to determine the clinical efficacy and safety of omalizumab for children with severe atopic eczema. This article describes the detailed statistical analysis plan for the ADAPT as an update to the published protocol and is submitted prior to knowing all outcomes.

**Method and design:**

The ADAPT is a randomised, double-blind, placebo-controlled trial with a primary objective to determine whether anti-IgE reduces eczema severity as assessed by the validated eczema score (objective SCORAD) after 24 weeks of treatment in children with severe eczema. This articles outline the overall analysis principles including considerations on sample definition in each analysis, missing data, and adjusted covariates. Comparability and representativeness of the randomised groups, primary and sensitivity analyses of the primary and secondary outcomes as well as subgroup analysis are described.

**Results:**

This prespecified statistical analysis plan has been developed to comply with international guidelines which will increase the transparency of the data analysis for the ADAPT.

**Trial registration:**

ISRCTN, identifier: ISRCTN15090567. Registered on 3 December 2014;

EU Clinical Trials Register, EudraCT Number: 2010-020841-29. Registered on 14 May 2010. The first participant was enrolled on 15 January 2015.

## Background

Eczema is a chronic inflammatory skin disorder with a lifetime risk of up to 22% of children by the age of 12–14 years. The available literature suggests that anti-immunoglobulin E (anti-IgE) may be of benefit in the treatment of eczema from at least the age of 7 years [1–4]. Studies and case reports to date have had small numbers of participants, have not been randomised or placebo controlled, or have included a heterogenous mix of participants of different ages.

The Atopic Dermatitis Anti-IgE Paediatric Trial (ADAPT) is a trial to determine the clinical efficacy and safety of omalizumab (Xolair, Novartis) for children with severe atopic eczema. Fuller details on the rationale and the design for the trial are given in the study protocol [5]; the prespecified statistical analysis plan (SAP) has been developed and finalised without knowing the treatment allocation or treatment-related study results.

This paper describes important features of the trial design and the statistical method and procedures which need to be adhered to and performed by the statistician responsible for this study. It should be read in conjunction with the protocol.

## Method and design

The ADAPT is a randomised, double-blind, placebo-controlled trial of anti-IgE therapy in children with severe eczema who have failed topical therapy. In total, 62 children aged 4–19 years are planned to be recruited within 18 months. Participants are eligible if they have severe eczema defined as an objective SCORing Atopic Dermatitis (SCORAD) score of over 40 at assessment (detailed exclusion and inclusion criteria are in the published protocol). Participants will be individually randomised in a 1:1 ratio to two treatment arms (omalizumab and matched placebo) using minimisation to ensure the balance of total IgE (≤1500 and >1500) and age (<12 and ≥12 years). The allocation will be performed by an online randomisation system hosted at the King’s Clinical Trials Unit (CTU).

## Primary objective

The primary objective of this study is to assess whether omalizumab will reduce eczema severity as assessed by the validated eczema score (SCORAD) after 24 weeks of treatment in children with severe eczema.

## Secondary objectives

The study will examine the influence of the study intervention on the rate of treatment failure, rate of alternative systemic therapy, quality of life, eczema severity as assessed by the Eczema Area and Severity Index (EASI), effect on co-existing allergic disease, number of eczema exacerbations, infective episodes of eczema, change in reactivity to food and aeroallergens and change in allergen-specific IgE. Detailed descriptions of the primary and secondary outcomes can be found in Table 1 as well as in the study protocol [5].

**Table 1:** Outcomes and analysis models

Outcome	Endpoints	Category	Analysis model
Primary			
Objective SCORAD	Difference in the objective SCORAD in both groups after 24 weeks of treatment	Continuous outcome measures	Mixed-effects linear regressions (primary) Instrumental variable regression
Secondary			
Treatment failure	Participants who have persistent severe eczema despite 2 courses of rescue therapy (0.5 to 1 mg/kg/day of orally administered prednisolone for a week at a maximum dose of 40 mg/day, followed by a week at 50% of this dose)	Binary outcome measures	Logistic regression model
Alternative systemic therapy	Requirement for alternative systemic therapy	Binary outcome measures	Logistic regression model
Eczema quality of life	• POEM • (C)DLQI	Continuous outcome measures	ANCOVA
Eczema severity	• Subjective SCORAD • EASI score	Continuous outcome measures	ANCOVA
Effect on co-existing allergic disease	PADQLQ	Continuous outcome measures	ANCOVA
Number of eczema exacerbations^c^	• Clinician-diagnosed exacerbation of eczema or • Increase on SCORAD by 15 points from last recorded SCORAD with participant/parent perception of worsening eczema	Numerical outcome measures	Poisson regression Negative binomial regression model Zero-inflated Poisson regression model (as appropriate)
Infective episodes of eczema^c^	Clinician-diagnosed and -treated infective episode of eczema, or clinically apparent, culture-positive infective exacerbations	Numerical outcome measures	Poisson regression Negative binomial regression model Zero-inflated Poisson regression model(as appropriate)
Allergen-specific IgE^a^	Change in allergen-specific IgE	Continuous outcome measures	ANCOVA
Reactivity to food and aeroallergens^a^	Change in skin-prick test reactivity to food and aeroallergens	Numerical outcome measures	Poisson regression Negative binomial regression model Zero-inflated Poisson regression model
Safety			
Adverse events^b^	Spontaneously reported AE will be collected throughout the follow-up period	Binary outcome	Descriptive analysis
Urea and electrolytes, creatinine, FBC, eosinophils, LFT, IgE, vitamin D, iron level, bone profile	Surveillance tests where abnormal ranges are defined using the ranges specified by the processing laboratory	Binary outcome	Descriptive analysis

^a^Only collected at screening and 24 weeks of treatment. The remaining outcomes are collected at baseline, 4-weekly during the 24 weeks of treatment, 36 weeks and 48 weeks

^b^Blood test and urine samples will be collected at baseline, 24 weeks, 36 weeks and 48 weeks. Clinical observations will be examined at every visit

^c^Chi‐square goodness‐of‐fit tests will be used to select the suitable model

*ANCOVA* analysis of covariance, *IgE* immunoglobulin E, *SCORAD* SCORing Atopic Dermatitis, *PADQLQ* Paediatric Allergic Disease Quality of Life Questionnaire, *(C)DLQI* (Children’s) Dermatology Life Quality Index, *EASI* Eczema Area and Severity Index, *AE* adverse events, *POEM* Patient-oriented Eczema Measure, *FBC* full blood count, *LFT* liver function test

## Sample size

Omalizumab is administered via subcutaneous injections which require fortnightly or monthly attendance at clinic to receive them. It is available from the manufacturers at an undisclosed cost in the United Kingdom. In order for omalizumab to be adopted into practice, a treatment effect that would make an important impact on the children’s quality of life would be required. Through discussion and consultation with the funder and clinicians, a relative reduction of around 33% in symptoms was selected to be the minimum important treatment effect to detect. Given the inclusion criteria the mean baseline SCORAD score is anticipated to be 45 and we aim to detect a change in SCORAD score of 13.5 points between the treatment arms. Based upon a study by Hindley [6] we assume that the standard deviation (SD) is 15, using a significance level of 5% with 90% power, and including a 15% dropout rate we aim to recruit 62 participants (31 each to each arm).

### Minimum Clinically Important Difference (MCID)

The study was powered to detect a minimum important treatment effect of a 13.5-point absolute change in objective SCORAD score taking into account the patient burden and high treatment cost. The MCID is the smallest difference in an outcome measure that represents a clinically relevant outcome to the patient, regardless of cost and burden. There is no verified MCID for SCORAD score in this severely affected paediatric population. In order to determine MCID, published studies have recommended the use of both anchor- and distribution-based methods [7]. A study by Schram et al. [8], which adopts an anchor-based approach, suggests that a MCID for the objective SCORAD score is 8.2. However, this is based on data from three RCTs on treatments for atopic eczema which included adults. The MCID reported by Schram et al. for children only, based on a subsample of *n* = 25, with an average age of 9.4 years, is 9.0. Since the patients included in the study by Schram et al. also had a milder baseline severity we employed a distribution-based method using data collected from the trial to calculate a MCID. Using the data from the first 47 ADAPT patients who completed week-24 assessments (75% of total sample size) adopting 0.7 SD of the change in score from baseline gives a MCID of 8.5. These MCIDs will be used to guide interpretation of the results from the primary analysis.

## General statistical principles

The principle of intention-to-treat (ITT) will be the main strategy of the analysis adopted for the primary outcome and all secondary outcomes. That is, all randomised participants will be analysed in the group randomised regardless of whether the allocated study treatments were received, or whether other interventions were received and regardless of any protocol deviations or violations [9]. A safety set (SS) population will consist of participants who receive at least one dose of allocated treatment, regardless of their eligibility for the study. The harm analyses will compare the harm outcomes between the two treatment groups in the safety population.

All regression analyses will include the minimisation variables (IgE (≤1500, >1500) and age (<10 or ≥10 years) as covariates. This is because adjustment for these stratification factors in the randomisation process will maintain correct type I error rates [10]. Additionally, for continuous outcomes, the outcomes measured at baseline will be included in regression analysis to increase power [11].

Any examination of subgroups, not specifically identified in the SAP, will be considered exploratory in nature and will be clearly identified. All *p* values will be two-sided and the significance level is set at 5% unless otherwise stated.

## Missing data

The number and proportion of participants missing objective SCORAD values by visit number will be tabulated. The primary analysis includes all observed data and assumes the probability that missing data is not dependent on the values of the unobserved data itself, conditional on the observed values of the variables included in the analysis model (missing at random (MAR) assumption). Sensitivity analysis will explore departures from the main MAR analysis assumption for all patients on the primary outcome using a pattern-mixture multiple imputation (MI) approach [12].

Imputation under MAR will initially be performed separately within each arm following the guidance suggested by White et al. [13]. The variables in the imputation model will be the same as those in the analysis model without including more auxiliary variables (e.g. predictors of missingness) after taking into account the relatively small sample size of this study [14]. Imputations will then be modified to reflect departures from the MAR assumption.

We will investigate the impact of a better or poorer response than that predicted by MAR (lower/higher objective SCORAD scores) for patients with missing data. Specifically, we define *δ* as the postulated mean difference in the rate of change of the objective SCORAD score between the observed and unobserved cases over 24 weeks, conditional on the variables in the imputation model. For each patient we then modify the MAR imputed observations accordingly by *δ*. Imputed data sets will be analysed using the primary analysis model. Results will be combined across imputed data sets using Rubin’s rules. We will repeat the analysis for a range of *δ*s corresponding to ±10, 20, 30, 40 and 50% of the rate of change of the objective SCORAD score observed over 24 weeks in all patients. We will also consider the possibility that data is missing informatively in one arm only and employ the outlined imputation approach separately by trial arm.

For baseline covariates, the amount of missing data is expected to be small. However, if this happens, in case of loss of power using observed data, mean values will be calculated from the non-missing values for the baseline variable using pooled data from both treatment groups [15]. With reference to those categorical variables, the imputed mean will be rounded up to nearest category level. This is justifiable because randomisation ensures that baseline scores are independent of treatment group and imputation keeps the statistical efficiency in the estimation of the treatment effect.

For those missing items within questionnaires, we firstly use the missing value guidance provided for questionnaires. If no guidance is provided we will then impute the missing values using the mean of the observed items within the same subscale if 20% or fewer items are missing. The scale score will be calculated based on the complete values and these replacements [16]. If more than 20% of items are missing in the questionnaire, multiple imputation will be used as discussed above.

## Statistical analysis

### Trial profile

**Fig. 1:**
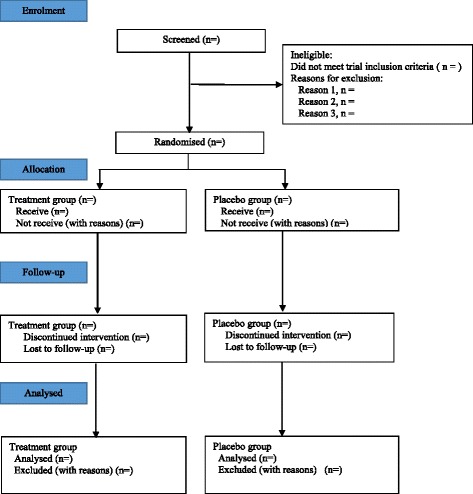
Consolidated Standards of Reporting Trials (CONSORT) trial flow chart for the Atopic Dermatitis Anti-IgE Paediatric Trial (ADAPT)

A Consolidated Standards of Reporting Trials (CONSORT) flow chart will be constructed (see Fig. 1). This will include the number of eligible patients, the number of patients agreeing to enter the trial, the number of participants withdrawing and lost to follow-up, the number continuing through the trial, and the number included in the analyses.

### Comparability/representativeness of randomised groups

All baseline descriptive variables of participants will be summarised by treatment arm. Continuous data will be expressed as *N*/mean/SD/min/Q1 (lower quartile)/median/Q3 (upper quartile)/max. Tabulations of frequencies for categorical data will include all possible categories and will display the number of observations in a category as well as the percentage (%) relative to number of available values within the respective treatment group unless otherwise specified. The number of missing values is reported for both types of variables. This will allow a visual assessment of whether the randomisation procedure succeeded in producing comparable arms, and tests of statistical significance will not be undertaken between arms at baseline; rather the clinical importance of any imbalance will be noted.

The number of participants who receive the injection outside the planned visit window of 5 days or more will be reported by visit number and treatment arm. Also, the mean cumulative dosage by planned dose will be plotted by treatment arm and separately for those receiving monthly and fortnightly injections.

### Descriptive statistics for outcomes

The distributions of all efficacy outcomes (in Table 1) will be presented in histograms (continuous/count) or bar charts (ordinal/binary) both overall and by group at each assessment point. A single table will be outputted with summary statistics for all outcomes by group and visit point. Furthermore, summary statistics will be plotted by line graphs for each outcome across time by intervention. Only participants with a completely recorded outcome will be used to calculate the summary measures.

### Analysis of primary efficacy outcome

#### Primary analysis

A linear mixed model will be used to obtain an estimate for the mean difference in objective SCORAD scores between the two treatment groups. Participant will be included as a random intercept (investigating adding a random slope on time), and time (investigating the possibility of linearising this effect across 8, 12, 16, 20 and 24 weeks), time-by-group interaction, baseline objective SCORAD score, IgE (≤1500, >1500) and age (<10 or ≥10 years) as fixed effect. An overall treatment effect for objective SCORAD score at 24 weeks will be estimated.

The response *y*_*ij*_ is the objective SCORAD score measurement for patient *i* at time *t*_*j*_. Both random intercept model (a) and random intercept and slope model (b) will be fitted as specified below:

- $$ \begin{array}{l}{Y}_{i j}={\beta}_0+{\beta}_1 TR{T}_i+{\beta}_2 SCORA{D}_i^0+{\beta}_3 Ig{E}_i+{\beta}_4 Ag{e}_i+{\beta}_5{t}_{12}+{\beta}_6{t}_{16}+{\beta}_7{t}_{20}+{\beta}_8{t}_{24}+\\ {}\kern2em {\beta}_9{t}_{12}* TR{T}_i+{\beta}_{10}{t}_{16}* TR{T}_i+{\beta}_{11}{t}_{20}* TR{T}_i+{\beta}_{12}{t}_{24}* TR{T}_i+{b}_i+{e}_{i j}\end{array} $$
- $$ \begin{array}{l}{Y}_{i j}={\beta}_0+{\beta}_1 TR{T}_i+{\beta}_2 SCORA{D}_i^0+{\beta}_3 Ig{E}_i+{\beta}_4 Ag{e}_i+{\beta}_5{t}_{12}+{\beta}_6{t}_{16}+{\beta}_7{t}_{20}+{\beta}_8{t}_{24}+\\ {}\kern2em {\beta}_9{t}_{12}* TR{T}_i+{\beta}_{10}{t}_{16}* TR{T}_i+{\beta}_{11}{t}_{20}* TR{T}_i+{\beta}_{12}{t}_{24}* TR{T}_i+{b}_{1 i}+{b}_{2 i}{t}_j+{e}_{i j},\end{array} $$

where *j* = time points (8, 12, 16, 20 and 24 weeks), *i* = participants,

*TRT*_*i*_: dummy variable (*TRT*_*i*_ = 0 or 1) of patient *i*,

*IgE*_*i*_: dummy variable for IgE (=0 or 1) of patient *i*,

*SCORAD*_*i*_^0^: baseline SCORAD score of patient *i*,

*Age*_*i*_: dummy variable for age (<10 or ≥10 years) of patient *i*,

*t*_*xx*_: dummy variable for time (= 0 or 1) at time point xx weeks.

Where *b*_*i*_ and *b*_1*i*_ are random intercepts, *b*_2*i*_ is random slopes, both *e*_*ij*_ and *b*_1*i*_, following normal distributions. An unstructured covariance matrix will be used. Models will be fitted using residual maximum likelihood (REML). The estimated treatment effect at 24 weeks, *β*_1_ + *β*_12_, will be reported with 95% confidence intervals and corresponding *p* value.

Model (a) will be the primary analysis model unless there is strong evidence for mis-specification of the model. The random slope model is less restrictive and possibly more realistic in its assumptions, i.e. the objective SCORAD score trajectories for each individual starting from a different level and following a different trend with a different slope. The primary interest is in determining whether *β*_1_ + *β*_12_ is significant and whether this varies between the two models (a and b).

The conclusion of the trial will be based on this analyses.

#### Planned sensitivity analyses

To investigate the robustness of the results of the primary analysis we will undertake a number of sensitivity analysis:

1. Subsequent adjustment for cumulative use of potent topical steroids (continuous variable) at 24 weeks, alternative systemic therapy (yes/no) at 24 weeks, rescue medication (yes/no) at 24 weeks based on the primary model
2. An analysis of Complier Average Causal Effect (CACE) by a two-stage least squares instrumental variable regression would be performed for the primary endpoint as analysis based upon ITT may underestimated the effect of actually receiving the treatment [17]. Here, we defined ‘compliers’ as those who complete more than 50% of injections (that is injections received relative to injections planned for the 24-week study period in groups). Randomisation will be used as an instrumental variable for treatment received with the same covariates in primary analysis models.

### Analysis of secondary efficacy outcomes

All analyses for secondary efficacy outcomes will be based on the ITT population and defined at week 24 unless specified otherwise. The missing data will be tackled according to the strategies mentioned above.

For each secondary outcome, we will adjust for the minimisation variables IgE (≤1500, >1500), age (<10 or ≥10 years) and baseline data (as appropriate).

Treatment failure (binary) and alternative systemic therapy (binary) will be analysed using a logistic regression model. The estimated treatment effect (odds ratio) will be reported with 95% confidence intervals and corresponding *p* value. Subjective SCORAD, EASI, Patient-Oriented Eczema Measure (POEM), Paediatric Allergic Disease Quality of Life Questionnaire (PADQLQ), (Children’s) Dermatology Life Quality Index ((C)DLQI) scores and allergen IgE levels will be analysis using analysis of covariance (ANCOVA). The estimated treatment effect (mean difference) will be reported with 95% confidence intervals and corresponding *p* value. The number of skin-prick test reactivities, infective episodes of eczema count, and number of eczema exacerbations will be analysed by Poisson regression, Negative binomial regression or zero-inflated Poisson regression models after checking the distribution of the dependent variable by Pearson chi‐square goodness‐of‐fit tests will ensure the selection of the correct statistical model. The estimated treatment effect (odds ratio) will be reported with 95% confidence intervals and corresponding *p* value. A summary of models for each of the outcomes can be found in Table 1.

### Analysis of safety outcomes

Information on adverse events (AE) will be collected by means of spontaneous reports from patients and carers, clinical observation and clinical examinations and blood tests. Adverse events will be coded using terms chosen by the clinical investigators with reference to the Medical Dictionary for Regulatory Activities (MedDRA) at the Preferred Term level. Abnormal ranges for blood tests will be defined using the ranges specified by the laboratory processing the sample.

Adverse events will be tabulated overall by severity and type (AE, adverse reaction, unexpected adverse reaction, serious AE, serious adverse reaction or unexpected serious adverse reactions). These will be summarised over the 48-week follow-up period and, where appropriate, by time of occurrence.. The numerator will indicate the number of affected participants at each time point from the SS population. The denominators will show how many participants were in the trial at the corresponding time point. If appropriate, the difference in proportion (95% confidence interval) will be estimated and time-to-event curves by treatment arm will be plotted. All AE will be listed individually.

### Subgroup analyses

A subgroup analysis is planned to investigate whether intervention effects differ between adherence, defined as the injections received relative to the injections planned for the 24-week study period in groups (≤50%, >50%; ≤75%, >75%; ≤90%, >90%). All subgroup analyses will be analysed using the same method as for the primary outcome. The results will be displayed by means of a forest plot.

### Software

Data management: an online data collection system for clinical trials (MACRO; InferMed Ltd.) will be used. This is hosted on a dedicated server at Kings’ Clinical Trial Unit. The CTU data manager will extract data periodically as needed and provide these in comma separated (.csv) format.

Statistical analysis: analysis will be performed using statistical software Stata, R or SAS.

### Tables and figures

The SAP describes the conventions to be used for presenting results in text and in tables and figures. Those conventions are based on the International Conference on Harmonisation (ICH) guideline for reporting clinical trial results. The planned tables are:

- Analysis population by study centre and treatment group
- Withdrawals, protocol deviations and violations by treatment group
- Baseline demographic and clinical characteristics by treatment group
- Baseline blood/urine investigations by treatment group
- Descriptive analysis for primary efficacy outcomes by treatment group across study visits
- Inferential analysis for primary efficacy outcomes by treatment group
- Sensitivity analysis for primary efficacy outcomes by treatment group
- Secondary efficacy outcomes by treatment group
- AE by treatment group across study visits

The planned figures are:

- Flow chart of participants through the study
- Bar chart/histogram for efficacy outcome over time by overall and treatment group
- Linear graph for efficacy outcome over time by treatment group
- Forest plot of effect of treatment on primary for all participants and for prespecified subgroups

This article presents the SAP for the ADAPT and should be read in conjunction with the trial protocol [5].

## Abbreviations

(C)DLQI: (Children’s) Dermatology Life Quality Index
AE: Adverse events
ANCOVA: Analysis of covariance
CACE: Complier Average Causal Effect
CTU: Clinical Trials Unit
EASI: Eczema Area and Severity Index
ITT: Intention-to-treat
MAR: Missing at random
PADQLQ: Paediatric Allergic Disease Quality of Life Questionnaire
POEM: Patient-oriented Eczema Measure
SAP: Statistical analysis plan
SCORAD: SCORing Atopic Dermatitis
SD: Standard deviation
SS: Safety set
